# A Case of Emphysematous Gastritis Managed With Conservative Treatment

**DOI:** 10.7759/cureus.84117

**Published:** 2025-05-14

**Authors:** Ashwin Jagadish, Zain Alabdin Ibrahim, Stephen Bakeler, Usama Abu-Heija, Shahnawaz Notta, Noah Hall

**Affiliations:** 1 Internal Medicine, East Tennessee State University James H. Quillen College of Medicine, Johnson City, USA; 2 Gastroenterology, East Tennessee State University James H. Quillen College of Medicine, Johnson City, USA; 3 Gastroenterology, James H. Quillen Veterans Affairs Medical Center, Johnson City, USA

**Keywords:** computed tomography, conservative treatment, emphysematous gastritis, gastroenterology, internal medicine

## Abstract

Emphysematous gastritis is a rare condition associated with a high mortality rate. It is associated with various medications, chronic medical problems, and ingestions. Individuals can present with abdominal symptoms and even hemodynamic changes. Evaluating the patient with computed tomography (CT) imaging can show inflammation of the gastric wall and intramural gas. A conservative approach is often used to treat emphysematous gastritis, although surgical intervention may be necessary. Our case involves a 67-year-old male patient who presented with acute encephalopathy and bradycardia. He was noted to have epigastric tenderness, so non-contrast CT imaging of the abdomen and pelvis was performed. The imaging revealed emphysematous gastritis. Our patient was successfully managed with intravenous antibiotics, intravenous fluids, proton-pump inhibitors, and nasogastric decompression. His encephalopathy and other symptoms improved while inpatient, and he was discharged in stable condition.

## Introduction

Emphysematous gastritis is a rare condition characterized by diffuse inflammation of the gastric wall and intramural gas [1]. There are less than 200 documented cases of emphysematous gastritis [2]. The condition can have a mortality rate of up to 60% [3]. There are many risk factors associated with emphysematous gastritis [1]. Prior to the year 2000, surgical intervention was the main treatment approach; however, there has been a trend toward conservative management since then [4]. We present a case of emphysematous gastritis that was successfully managed with conservative treatment.

## Case presentation

A 67-year-old male patient with a history of coronary artery disease, non-insulin-dependent type 2 diabetes mellitus, hypertension, hyperlipidemia, atrial fibrillation on apixaban, and cirrhosis due to alcohol presented to the emergency department due to acute encephalopathy and multiple ground-level falls. He was somnolent and was unable to provide a complete history. Per his spouse, the patient's encephalopathy started the previous night and was progressively worsening. He also had multiple ground-level falls that were triggered by positional changes. There was no recent consumption of alcohol, corticosteroids, or non-steroidal anti-inflammatory drugs.

**Figure 1:**
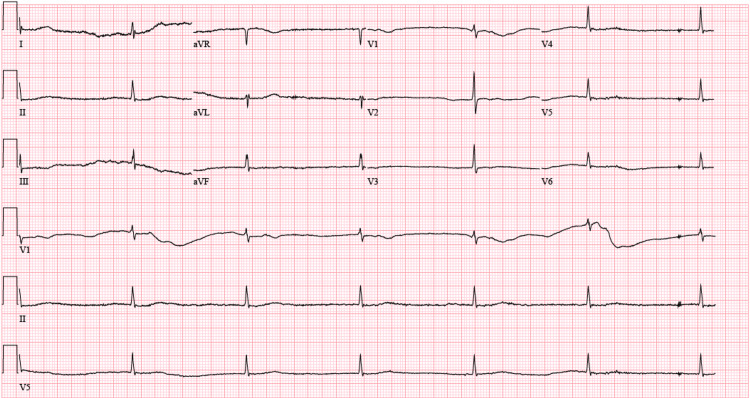
Electrocardiogram Electrocardiogram showing bradycardia.

On presentation, his blood pressure was 99/57 mmHg, his heart rate was bradycardic at 37 beats per minute, and his respirations were 18 breaths per minute. His electrocardiogram showed junctional bradycardia (Figure 1). His physical examination was notable for dry mucous membranes, diminished breath sounds at the bases, bradycardia, and altered mentation. His abdomen was soft, nontender, and nondistended; bowel sounds were present in all quadrants. Additionally, asterixis was absent. His initial laboratory results are presented in Table 1. His initial chest radiograph did not reveal any acute cardiopulmonary process. His heart rate did not improve adequately with atropine and 2 L of normal saline, so a dopamine infusion was initially required.

**Table 1:** Initial laboratory results

Parameters	Result	Reference Range
White Blood Cells (cells/µL)	7,600	4,800–10,500
Red Blood Cells (cells/µL)	4,300,000	4,400,000–5,600,000
Hemoglobin (g/dL)	12.9	13.6–17.3
Hematocrit (%)	39.4	39.5–51.7
Mean Corpuscular Volume (fL)	92.3	83.5–96.8
Mean Corpuscular Hemoglobin (pg)	30.2	27.3–33.3
Mean Corpuscular Hemoglobin Concentration (g/dL)	32.7	32.9–34.6
Red Blood Cell Distribution Width (%)	14.1	11.6–14.1
Platelets (cells/µL)	160,000	166,000–383,000
Mean Platelet Volume (fL)	10.7	6.5–10.5
Sodium (mEq/L)	134	137–145
Potassium (mEq/L)	4.7	3.6–5.2
Chloride (mEq/L)	94	98–107
Bicarbonate (mEq/L)	29	22–29
Glucose (mg/dL)	180	70–99
Blood Urea Nitrogen (mg/dL)	40	5.0–25.0
Creatinine (mg/dL)	2.5	0.7–1.3
Protein, Total (g/dL)	7.5	6.3–8.2
Albumin (g/dL)	4.3	3.5–5.0
Calcium (mg/dL)	9.7	8.4–10.4
Total Bilirubin (mg/dL)	0.5	0.2–1.3
Alkaline Phosphatase (U/L)	121	40–150
Aspartate Aminotransferase (U/L)	41	5–34
Alanine Aminotransferase (U/L)	55	0–55
Thyroid Stimulating Hormone (µIU/mL)	0.843	0.35–4.94
Ammonia (µmol/L)	21	18–72
Creatine Phosphokinase (U/L)	34	30–200

**Figure 2:**
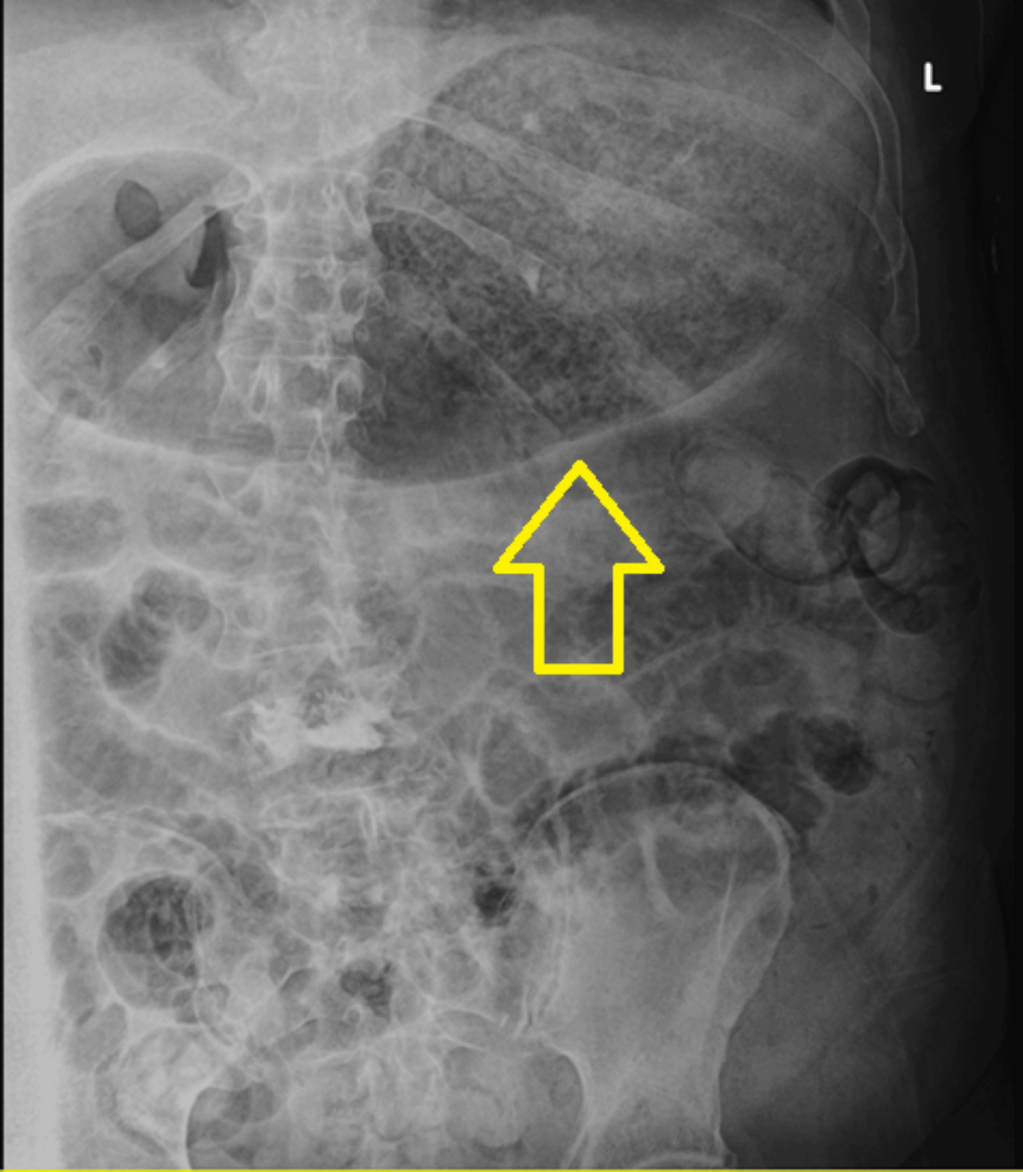
Abdominal radiograph demonstrating gastric distention The arrow indicates the gastric distention.

**Figure 3:**
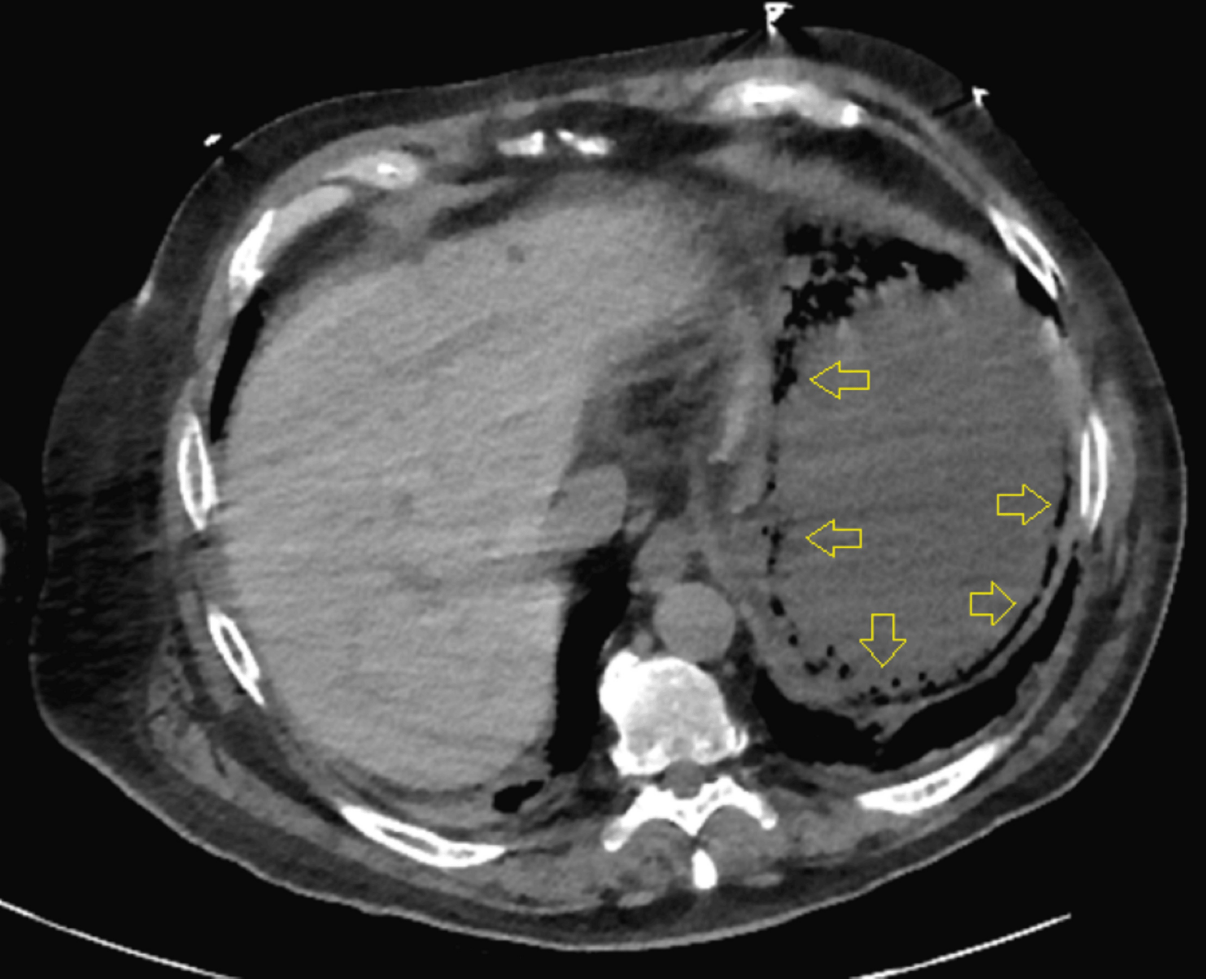
Initial computed tomography imaging demonstrating emphysematous gastritis The arrows indicate the emphysematous gastritis.

The following day, abdominal tenderness was observed during the physical examination. The initial abdominal radiograph revealed gastric distention (Figure 2). Subsequent computed tomography (CT) evaluation of the chest, abdomen, and pelvis without contrast revealed emphysematous gastritis (Figure 3). He was started on 3.375 g of intravenous piperacillin-tazobactam every six hours. He was also started on 40 mg of pantoprazole twice daily. A nasogastric (NG) tube was placed, and more than 1 L of fluid was removed. A temporary pacemaker was also placed.

**Figure 4:**
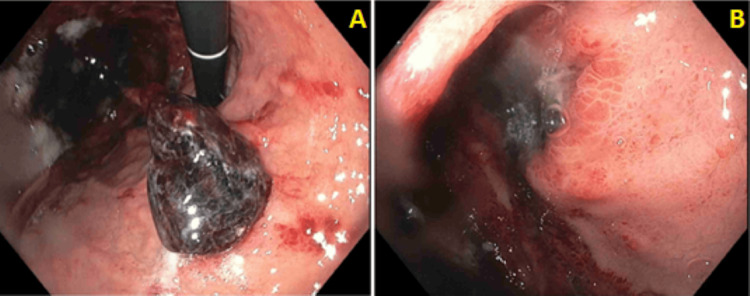
Results from esophagogastroduodenoscopy (A) Gastric body; (B) gastric fundus.

**Figure 5:**
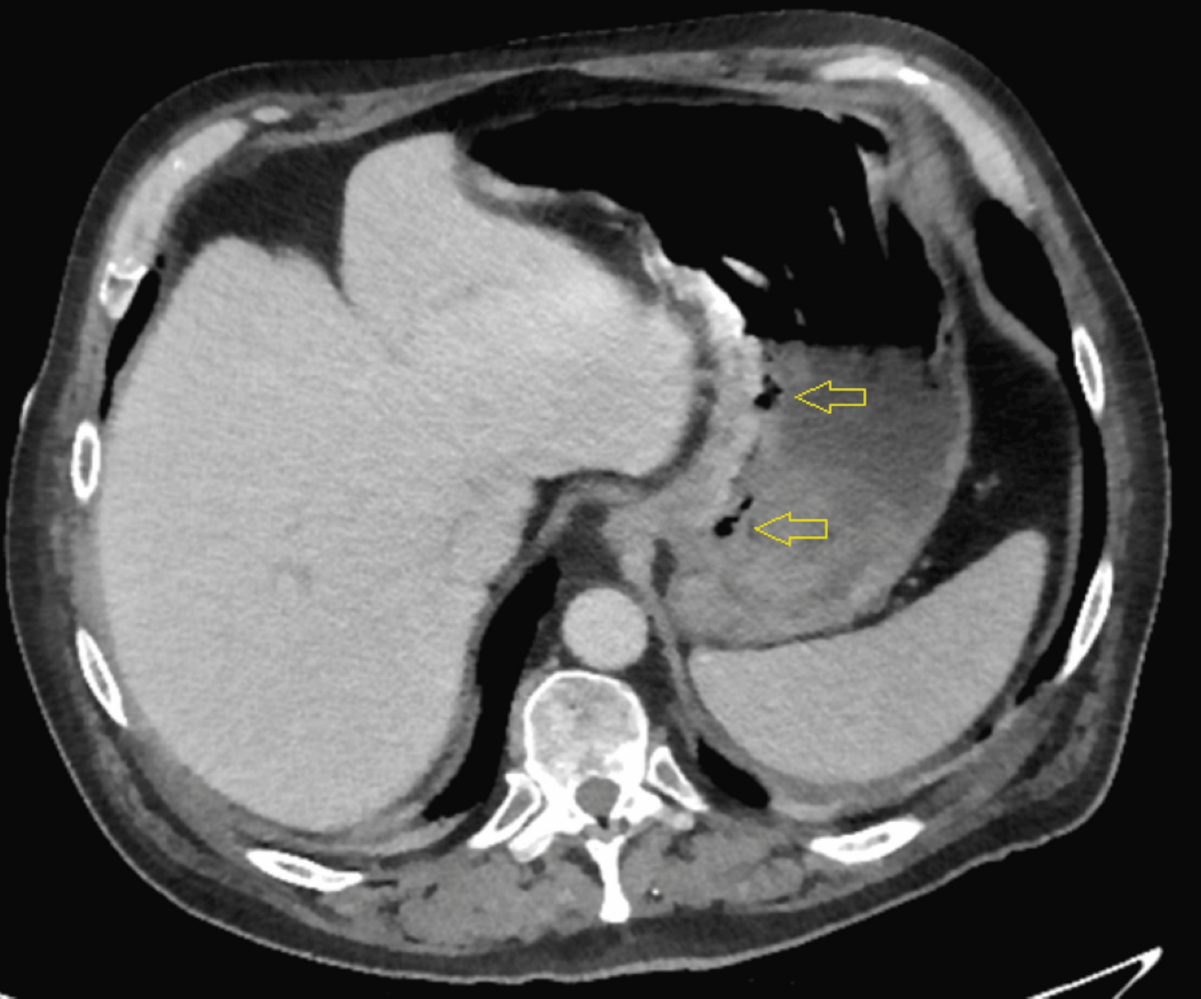
Repeat computed tomography imaging demonstrating improving emphysematous gastritis The arrows indicate the improving emphysematous gastritis.

Two days later, his leukocyte count peaked at 13,200 cells/µL. Ongoing evaluation of the NG tube contents revealed bloody material. He subsequently underwent esophagogastroduodenoscopy (EGD) three days after the NG tube placement. The procedure revealed multiple ulcers in the gastric cardia and fundus that were covered by clots (Figures 4A, 4B). Given these findings, the EGD was terminated, and biopsies were not obtained. A repeat CT of the abdomen and pelvis with contrast, completed soon after the EGD, showed improving emphysematous gastritis (Figure 5). The NG tube was subsequently removed. During the interval between NG tube insertion and removal, the patient required maintenance fluids amounting to 6 L.

The following day, the temporary pacemaker was removed after improvement of the bradycardia. The cardiology team determined that the patient did not require permanent pacemaker placement while inpatient. Over the rest of his hospitalization, his encephalopathy ultimately resolved. Blood cultures, which were obtained upon presentation, remained negative throughout the hospitalization. He completed a six-day course on antibiotics, per infectious diseases recommendations. He was stable at discharge and was advised to continue the pantoprazole. Outpatient gastroenterology and cardiology follow-up appointments were also recommended.

## Discussion

Emphysematous gastritis is a rare condition characterized by gas in the gastric wall and can have a mortality rate of up to 60% [3]. It is important to differentiate emphysematous gastritis from gastric emphysema [4]. Gastric emphysema is generally considered benign and results from air being introduced into the gastric wall [4]. On imaging, intramural gas tends to appear round, whereas intramural gas is more linear in emphysematous gastritis [4]. Management of gastric emphysema is often conservative, and the condition often resolves spontaneously [4].

Emphysematous gastritis can be associated with medications, chronic medical conditions, and ingestions [5]. Categories of medications associated with the condition include immunosuppressive agents, non-steroidal anti-inflammatory drugs, and corticosteroids [5]. Medical conditions associated with emphysematous gastritis include diabetes mellitus, ethanol consumption, renal disease, and poor nutrition [5]. Consumption of corrosive material is also associated with the condition [6]. Causative organisms have been determined in approximately half of the cases [7]. In cases where an organism is identified, the most common species of organisms include *Klebsiella*, *Candida*, *Clostridium*, *Streptococcus*, and *Pseudomonas* [4]. As compared to patients with gastric emphysema, patients with emphysematous gastritis tend to show signs of severe illness, such as sepsis [4]. In our case, the patient's diagnosis was based on the severity of presentation and intramural gas appearance on imaging.

Symptoms of emphysematous gastritis can include pain in the epigastric region, nausea, emesis, loose stools, leukocytosis, and hemodynamic instability [8]. Various imaging modalities can be used to help diagnose emphysematous gastritis [3]. Abdominal radiographs can show gastric dilation with intramural gas [3]. However, CT scans are often used since they are more sensitive [3]. Pertinent imaging findings for emphysematous gastritis include thickening of the gastric wall and the presence of intramural, irregular, mottled gas [9].

Management of emphysematous gastritis involves both medications and supportive measures [5]. Intravenous fluids, along with antibiotics that have coverage against gram-negative and anaerobic organisms, are often necessary [5]. Additionally, an NG tube can be placed to assist with gastric decompression [10]. Surgical intervention may be required if the patient's status declines or there are concerns for peritonitis, infarction of the stomach, or inadequate response to medical management [11].

## Conclusions

Emphysematous gastritis is an uncommon condition with a mortality rate of up to 60%. It can be associated with medications, chronic medical problems, or ingestions. In approximately 50% of cases, a causative organism is identified. It is often diagnosed using imaging, with CT scans being more sensitive than radiographs. Management of the condition is generally supportive care alongside antibiotic coverage. However, in some cases, surgical intervention may be necessary.
